# Establishment and Application of a Dual-Labeling Time-Resolved Fluorescence Immunoassay Method for Simultaneous Detection of the Troponin I-C Complex and Full-Size-Troponin I

**DOI:** 10.3389/fcvm.2020.596051

**Published:** 2021-01-14

**Authors:** Biao Huang, Jian Wu, Hao Chen, Li Zhang, Xiumei Zhou, Qingqing Wu, Ting Li, Yigang Wang, Penguo Xia, Yaping Dai, Guoyin Kai, Pengfei Liu, Hao Pei

**Affiliations:** ^1^Immounoassay Laboratory, College of Life Sciences and Medicine, Zhejiang Sci-Tech University, Hangzhou, China; ^2^State Key Laboratory for the Diagnosis and Treatment of Infectious Diseases, National Clinical Research Center for Infectious Diseases, The First Affiliated Hospital, College of Medicine, Zhejiang University, Hangzhou, China; ^3^Department of Laboratory Medicine, The First People's Hospital of Yancheng City, Yancheng, China; ^4^Clinical Lab, Wuxi No.5 People's Hospital, Wuxi, China; ^5^Chinese Medicine Resources Teaching and Research Laboratory, College of Pharmacy, Zhejiang Chinese Medical University, Hangzhou, China; ^6^Department of Gastroenterology, The Jiangyin Clinical College of Xuzhou Medical University, Jiangyin, China

**Keywords:** cardiac troponin I, acute myocardial infarction, dual-labeled, immunoassay, time-resolved fluorescence

## Abstract

**Background:** The measurement of cardiac troponin I (cTnI) is widely used in the diagnosis of acute myocardial infarction (AMI). Although existing cTnI detection methods measure total cTnI, the significance of undegraded full-size-cTnI levels is still not well-understood. In this study, we have established a novel dual-labeling time-resolved fluorescence immunoassay (TRFIA) technique that simultaneously detects the cTnI-C complex and full-size-cTnI, allowing us to explore the clinical value of full-size-cTnI determination.

**Methods:** An antibody against the 23–43 amino acid region of cTnI protected by endogenous cTnC is coupled to magnetic beads to provide a solid-phase antibody for capturing all cTnI. An antibody against cTnC in the cTnI-C complex labeled with Eu^3+^ was used to detect the cTnI-C complex, and an antibody labeled with Sm^3+^ near the C-terminal 190–203 amino acids of cTnI was used to detect full-size-cTnI. Through dual-labeling TRFIA, cTnI-C complex, full-size-cTnI, and the full-size-cTnI/cTnI-C ratio can be detected simultaneously. The dual-labeling TRFIA technique was used to analyze serum samples collected at different times during treatment and compare their full-size-cTnI/cTnI-C ratios.

**Results:** The sensitivity for the cTnI-C-TRFIA complex was 0.02 ng/mL, the measurement range was 0.02–40 ng/mL, the average intra-batch coefficient of variation (CV) was 4.35%, and the inter-average CV was 6.23%. The correlation coefficient between cTnI-C-TRFIA and commercial cTnI-CLIA methods was *R*^2^ = 0.8887. The sensitivity for full-size-cTnI-TRFIA was 0.04 ng/mL, the measurement range was 0.04–40 ng/mL, the average intra-batch CV was 4.95%, and the average inter-batch CV was 7.79%. The correlation coefficient between full-size-cTnI-TRFIA and commercial cTnI-CLIA methods was *R*^2^ = 0.7247.

**Conclusions:** Dual-labeling full-size-cTnI/cTnI-C-TRFIA analysis is helpful for determining the length of time of chest pain before admission and the degree of continuous release of cTnI in the myocardium. Thus, it is more for early prognosis than just detecting cTnI.

## Introduction

Cardiac troponin (cTn) is a regulatory protein of myocardial muscle contraction. cTn is composed of three different subunits: cardiac troponin I (cTn I), troponin C (TnC), and cardiac troponin T (cTnT). cTn is present in myocardial cells in the form of the cTnI-C-T complex and free cTnI, which is released into blood circulation when acute myocardial infarction (AMI) occurs. Then, cTnI-C-T can be further decomposed into the cTnI-C complex and free cTnT, where the cTnI- C complex is the main form in the blood (1). Their levels are minimal in normal serum and are increased significantly upon AMI. Although the half-life of troponin is very short (cTnT, 2 h; free cTnI 2 h−5 d), the process of its degradation from myofibrils takes a long time. Thus, it can persist in the bloodstream. Accordingly, cTnI and cTnT can be used as markers of myocardial cell death and are used to assist in the diagnosis of AMI (2, 3). The total length of cTnI is 210 amino acids, of which the 30–110 amino acid region is protected by cTnC and is thus the most stable (4). The N- and C-termini of cTnI are partially hydrolyzed by endogenous proteases due to the lack of cTnC protection, especially after 20 h of the onset of clinical characterization. At present, cTnI detection mostly detects the 30–110 amino acid region protected by cTnC to represent cTnI. In addition, because the main form of cTnI in the blood is the binary complex form cTnI-TnC, free cTnI exists only in trace amounts or cannot be detected at all (5). cTnC is not affected by phosphorylation, its proteolysis does not readily occur, and it is not sensitive to autoantibodies and heparin in the sample (1). One cTnC-specific antibody and one cTnI-specific antibody can be paired to detect the cTnI-C complex. Since there is only one cTnI fragment on a cTnI-C complex, the concentration of the cTnI-C complex is equivalent to the concentration of cTnI, i.e., the concentration of the cTnI-C complex represents the total cTnI concentration in serum, which helps to improve the analytical sensitivity and repeatability of cTnI detection (6–8). Although existing cTnI detection methods measure total cTnI, which can be a good indicator in the diagnosis of AMI, the significance of full-size-cTnI concentration is not well-understood.

**Figure 1:**
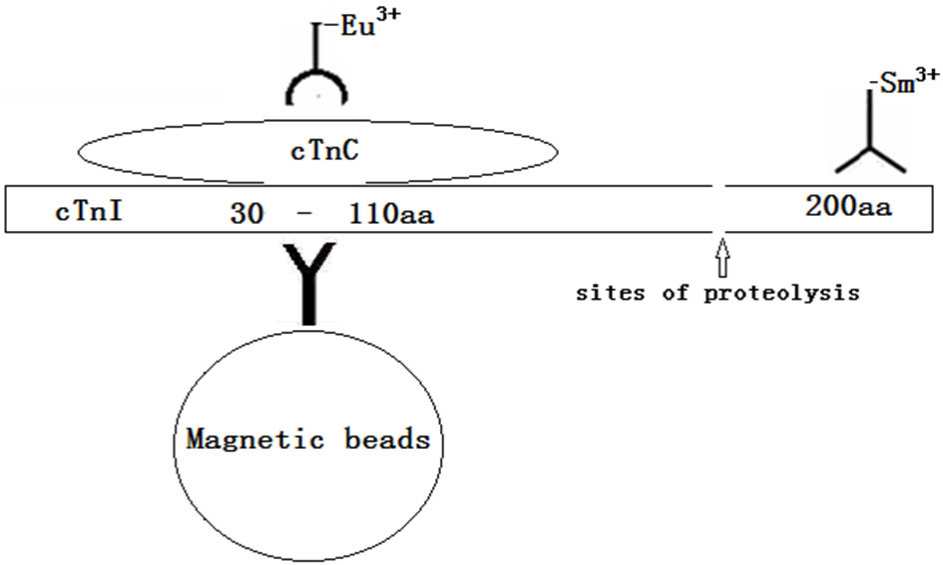
The reaction principle of simultaneously detects cTnI-C complex /full-size-cTnI-TRFIA.

In this study, we first established a dual-labeling time-resolved fluorescence immunoassay (TRFIA) that simultaneously detects the cTnI-C complex and full-size-cTnI to explore the clinical value of full-size-cTnI detection. The reaction principle is shown in Figure 1. An antibody against the 23–43 amino acid region of cTnI protected by endogenous cTnC is coupled to magnetic beads to provide a solid-phase antibody for capturing all cTnI. An antibody against cTnC in the cTnI-C complex labeled with Eu^3+^ was used to detect the cTnI-C complex, and an antibody labeled with Sm^3+^ near the C-terminal 190–203 amino acids of cTnI was used to detect full-size-cTnI (due to the epitope site being very close to the C-terminus, here it is termed full-size-cTnI). Since cTnI is easily hydrolyzed at the end after entering the blood, the full-size-cTnI concentration is decreased, and the cTnI-C complex is more stable. Through dual-labeling TRFIA, total cTnI, full-size-cTnI and the full-size-cTnI/cTnI ratio can be detected simultaneously.

## Materials and Methods

### Reagents and Equipment

Anti-cTnI-C complex antibody 20C6 and natural cTnI-C complex antigen were purchased from HyTest Ltd. Anti-cTnI amino acid 23–43 region as the capture antibody, anti-cTnI C-terminal 190–203 amino acid antibody C190, and β-naphthoyltrifluoroacetone (β-NTA) were provided by Zhejiang Boshi Biological Technology Co., Ltd. Amino-functionalized magnetic microspheres were purchased from Suzhou Beaver Biomedical Engineering Co., Ltd. Diethylenetriaminepentaacetate (DTPA), bovine serum albumin (BSA), Tris, and Triton X-100 were purchased from Sigma (St. Louis, MO, USA). Sepharose CL-6B columns were obtained from Pharmacia Co. (England). Ninety-six-well polystyrene microtiter plates were obtained from Xiamen Xinchuang Company (China). Eu- and Sm-labeling kits, were purchased from Perkin Elmer (USA). Pure water was produced by Barnstead Equipment. Other reagents used were of analytical reagent grade.

A DELFIA 1420 automatic immunoassay system (Perkin Elmer) was used to measure Eu^3+^ and Sm^3+^ fluorescence.

### Blood Samples

Serum was provided by the First Hospital of Yancheng City, Jiangsu Province according to the Helsinki Declaration on AMI patients (9). The studies involving human participants were reviewed and approved by The Ethics Committee of the First People's Hospital of Yancheng City (No. 2017044). Patients with AMI were diagnosed by clinical examination. The diagnosis was based on clinical phenomena, typical electrocardiogram changes, and elevated serum cTnI concentrations (a total of 69 cases were determined by the cTnI chemiluminescence method (CLIA) using a Merrill mini-VIDAS automatic fluorescence immunoanalyzer). Sixteen healthy subjects were used as a control group. Peripheral blood (5 mL) was collected from AMI patients 2 to 33 h after the onset of chest pain and the serum was separated. Among them, 15 patients were sampled 2–4 times at different times for monitoring.

### Preparation of cTnI Antibody Magnetic Microspheres

Magnetic microspheres with a diameter of 2 μm were activated with glutaraldehyde at a mass concentration of 5%. After mixing at room temperature for 4 h, they were washed with 0.05 mol/L PBS buffer (pH 7.2) three times, and then suspended in the same buffer solution. Then, 100 μL of the suspension was added to 1 mL of 0.05 mol/L PBS buffer (pH 7.2) containing 50 μg of capture antibody and incubated at 25°C for 2 h. Magnetic separation was used to isolate the magnetic particles. They were washed with 0.05 mol/L PBS buffer (pH 7.2) three times, blocked with 1 mL of PBS containing 5% BSA 0.01 mol/L (pH 7.2) for 30 min at 25°C, washed with 0.05 mol/L PBS buffer (pH 7.2) three times, and finally resuspended in 0.05 mol/L Tris-HCl buffer (pH 7.2) containing 0.5% BSA and 0.1% NaN_3_. The final suspension was stored at 2–8°C.

### Preparation of Labeled Antibodies

#### Sm-Labeled cTnI Monoclonal Antibody C190

cTnI antibody C190 (1 mg) was subjected to buffer exchange using a PD-10 column. The eluent was 50 M Na_2_CO_3_/NaHCO_3_ buffer (pH 8.5) containing 0.155 M NaCl, and the protein fraction was collected. Then, according to the Sm-labeling kit instructions, 0.2 mg of the samarium isothiocyanate benzyl diethylene triamine tetraacetate sodium was added to the cTnI monoclonal antibody solution, and the resultant mixture was magnetically stirred at 25°C for 20 h. The reaction solution was transferred to a Sepharose CL-6B column equilibrated with Tris buffer (80 mM, pH 7.8). The protein fraction was collected, diluted with 80 mM Tris buffer (pH 7.8) containing 0.5% BSA and 0.1% NaN_3_, and stored at 2–8°C.

#### Preparation of Eu-Labeled cTnC Monoclonal Antibody 20C6 Solution

cTnC antibody 20C6 (1 mg) was subjected to buffer exchange in the same manner as that used above, and 0.2 mg europium isothiocyanate benzyl diethylene triamine tetraacetate sodium was added, and the reaction solution was magnetically stirred at 25°C for 20 h and then subjected to chromatographic separation using a Sepharose CL-6B column. The protein fraction was collected, diluted with 80 mM Tris buffer (pH 7.8) containing 0.5% BSA and 0.1% NaN_3_ and stored at 2–8°C.

### Preparation of Other Reagents

The enhancement solution was prepared from 3.6 mL glacial acetic acid, 0.5 g sodium acetate, 0.05 g β-naphthoyl trifluoroacetone, 0.03 g trioctylphosphine oxide, and 1 mL Triton X-100, which were mixed well, diluted with deionized water to a volume of 1,000 mL, and stored at 2–8°C.

The reaction buffer was a 50 mM Tris-HCl buffer solution (pH 7.8) containing 8 mM NaCl, 0.1% BSA, 50 μmol/L DTPA, 0.1 ml/L Tween-80, and 0.1% NaN_3_;

The washing solution was a 50 mM Tris-HCl buffer solution at pH 7.8, containing 12.49 g/L NaCl, 1.11 g/L Tween-20.

### Preparation of Natural cTnI Calibrator Solution (Containing Full-Size-cTnI-C Complex)

A natural cTnI antigen solution at a concentration of 1 mg/mL was used to prepare standard solutions with different concentrations by series dilution with Tris-HCl reaction buffer (50 mmol/L pH 7.8) containing 0.2% BSA and 0.1% NaN_3_. The standards were stored at 2–8°C.

### Dual-Labeling TRFIA for Simultaneous Detection of Full-Size-cTnI and the cTnI-C Complex

First, a cTnI sample or standard solution (100 μL) was mixed with 50 μL of the magnetic cTnI monoclonal antibody particle solution. Then, 50 μL Sm-labeled cTnI monoclonal antibody C190 solution and 50 μL of Eu-labeled cTnC monoclonal antibody 20C6 solution diluted in reaction buffer were added to the mixed magnetic particle solution, which was then incubated at 37°C for 8 min and washed with washing solution eight times. Magnetic separation was then performed, then the enhancement solution was added. DELFIA_1420_ was used to measure the fluorescence of Eu and Sm in the calibration and sample solutions. Standard curves of the concentrations of cTnI-C complex and full-size-cTnI against fluorescence were used to calculate the concentrations of the analytes.

### Full-Size-cTnI/cTnI-C-TRFIA Methodology Evaluation

#### Detection Limit

The fluorescence values of the 10-well standard at zero calibrators were determined to obtain $\bar{x}$ plus two S.D. values, and the corresponding concentration of cTnI-C and full-size-cTnI on the standard curves were taken to be their respective sensitivities.

#### Correlation

Sixteen healthy samples and 69 samples from patients with different cardiovascular diseases were tested by both the TRFIA method and the conventional CLIA method, and the correlation between the results of the two methods was assessed.

#### Precision

The test samples were diluted to high, medium, and low concentrations, and the TRFIA method was used to test the samples and establish intra- and inter-batch precision.

#### Specificity

cTnT at a concentration of 20 ng/mL was used as a sample, and the concentrations of cTnI-C and full-size-cTnI in the sample were measured.

#### Statistical Analysis

SPSS 19.0 software (IBM, USA) was used for data analysis, and the *t*-test was used to compare the measurement data with normal distribution. The results are expressed as $\bar{x}$ ±*s*. Rank sum was used to compare the measurement data with non-normal distribution. Counting data was compared between the groups by χ^2^ testing. Linear correlation analysis was performed using Microsoft Excel. *P* < 0.05 was considered statistically significant.

## Results

### Full-Size-cTnI/cTnI-C-TRFIA

**Figure 2:**
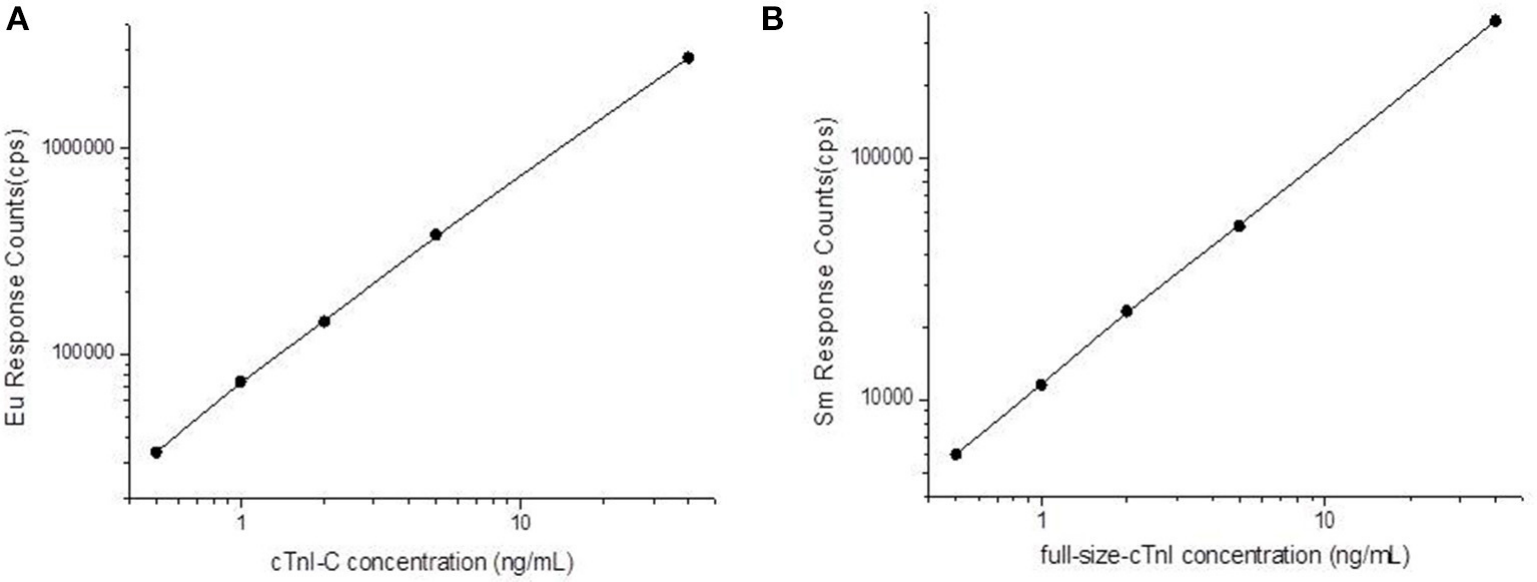
Full-size-cTnI/cTnI-C-TRFIA standard curves. **(A)** TRFIA standard curve of cTnI-C complex. **(B)** TRFIA standard curve of full-size-cTnI.

A double logarithmic function was used to process the standard curves shown in Figure 2. The sensitivity of TRFIA for the detection of the cTnI-C complex was found to be 0.02 ng/mL. The measurement range was 0.02–40 ng/mL, and the average intra-batch coefficient of variation (CV) for the high, medium, and low concentration samples was 4.35%, and the inter-average CV was 6.23%. The sensitivity of TRFIA for detecting full-size-cTnI was 0.04 ng/mL, and the measurement range was 0.04–40 ng/mL, the average intra-batch CV for the high, medium, and low concentration samples was 4.95%, and the average inter-batch CV was 7.79%. In the analysis of cTnT samples with a concentration of 20 ng/mL, the detection concentrations for the target analytes were lower than the sensitivity, indicating that the method has good specificity.

### Correlation With Conventional CLIA

**Figure 3:**
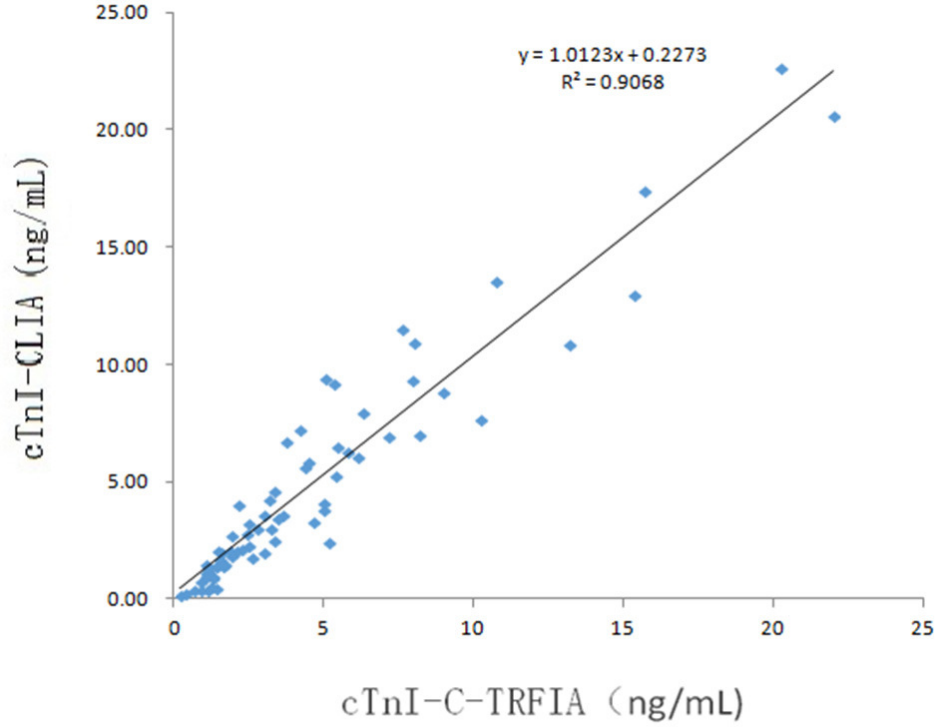
Method correlation for cTnI-C-TRFIA and cTnI-CLIA.

**Figure 4:**
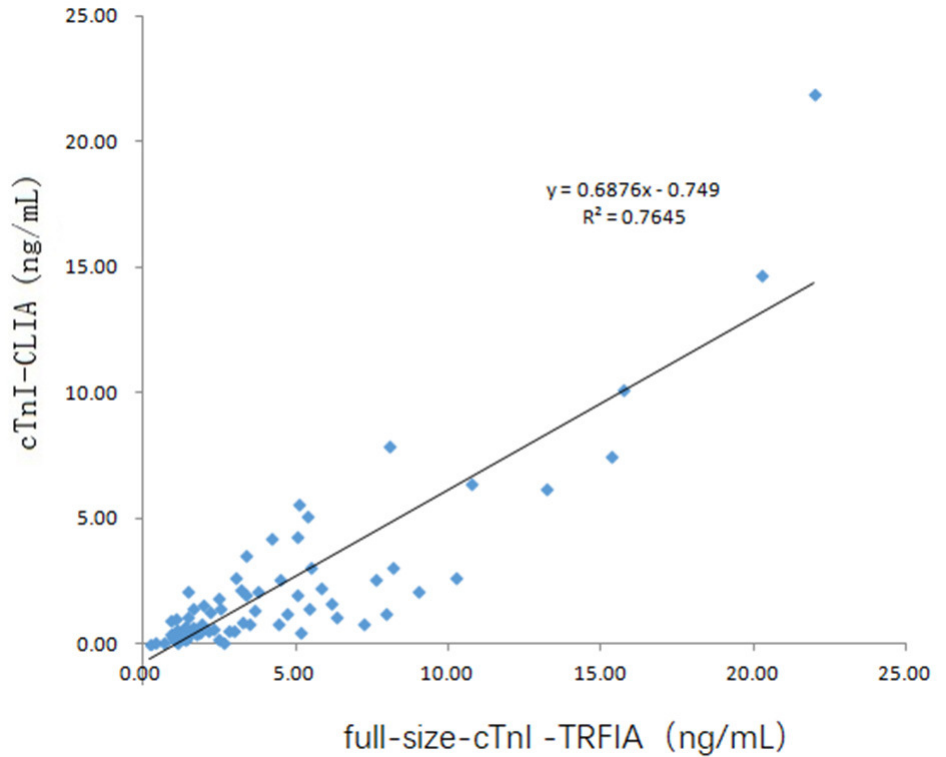
Method correlation for full-size-cTnI-TRFIA and cTnI-CLIA.

Serum samples from 16 healthy human subjects and 69 cardiovascular disease patients were analyzed using cTnI-TRFIA and cTnI-CLIA. Taking 0.11 ng/mL as the cut-off, the results for the 16 normal human sera were negative. Correlation analysis was performed on specimens within the detection range of the two methods. The correlation between the cTnI-C complex serum concentrations detected by the TRFIA method and the CLIA method are shown in Figure 3. The correlation coefficient between the two is *R*^2^ = 0.9068, indicating good correlation. Thus, the detection of cTnI-C complex by the TRFIA and cTnI-CLIA methods are consistent, and the concentration of cTnI-C complex can be used to express the concentration of cTnI. The sera of 69 patients with cardiovascular disease were tested by full-size-cTnI-TRFIA and cTnI-CLIA methods. The correlation between the results within the detection range of the two methods is shown in Figure 4. The correlation coefficient is *R*^2^ = 0.7645, indicating moderate correlation, and the value obtained by full-size-cTnI-TRFIA was generally lower than that obtained by CLIA.

**Figure 5:**
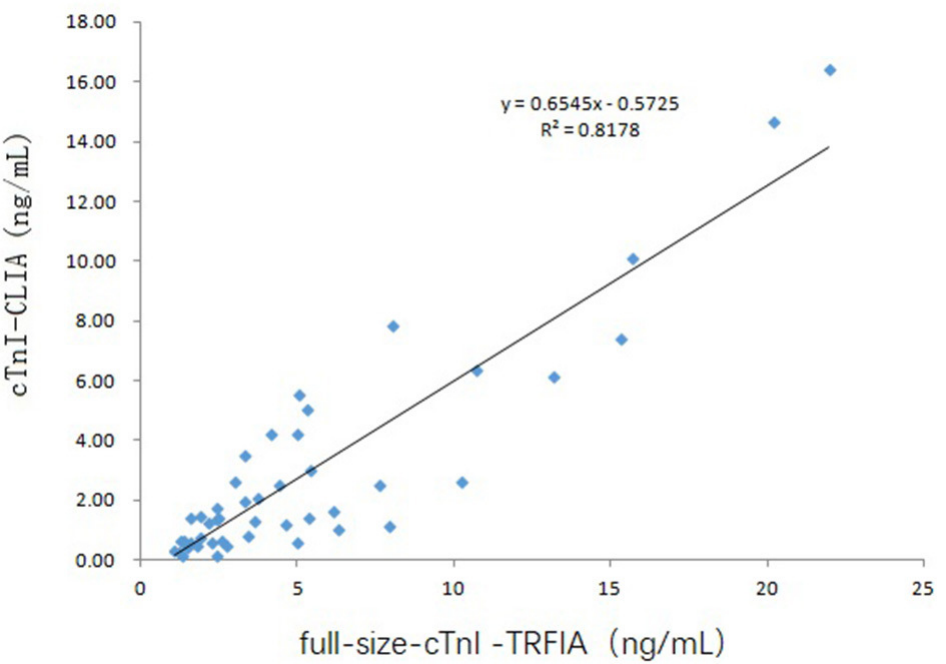
Correlation of full-size-cTnITRFIA and cTnI-CLIA results for first-diagnosed patients.

Since 22 of the 69 serum specimens were the 2nd to 4th collections within 2–10 days after consultation, these 22 specimens were not considered for correlation analysis. The correlation between serum full-size-cTnI-TRFIA results and cTnI-CLIA results for 47 first-diagnosed patients is shown in Figure 5. The correlation between full-size-cTnI-TRFIA and cTnI-CLIA is *R*^2^ = 0. 8178, and the correlation between the two methods is better than that when all 69 patients are considered.

**Figure 6:**
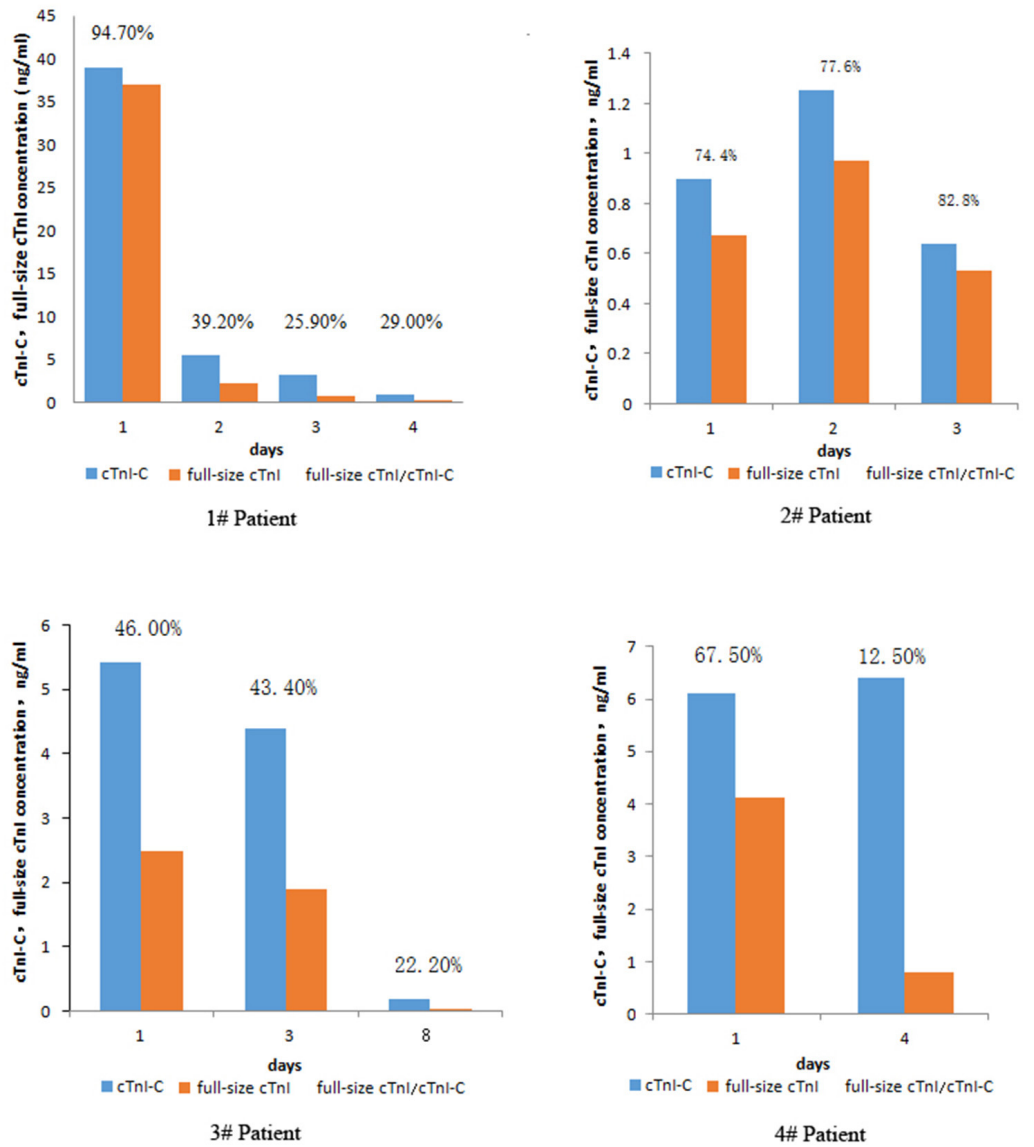
Changes in serum cTnI-C, full-size-cTnI concentration, and full-size-cTnI/cTnI-C ratio at different days during treatment of four patients.

In this retrospective study, a total of 18 patients had been subjected to serum sampling two times or more. One of them was sampled four times, two were sampled three times. In order to explore the diagnostic value of full-size-cTnI and cTnI analyses, we selected four patients for continuous multi-day follow-up testing, comparing the ratios of serum cTnI-C to full-size-cTnI collected at different times during treatment. The results are shown in Figure 6.

Patient 1, a 64-year-old male, was seen within 7 h of presenting chest pain. After admission, he was diagnosed with AMI and coronary heart disease. On the first day of admission, the patient's serum concentrations of full-size-cTnI and cTnI-C were very high, both exceeding 30 ng/mL. The full-size-cTnI/cTnI-C ratio was 94.7%, and surgery was performed immediately after consultation. Subsequent continuous cTnI concentration testing was carried out. The cTnI concentration was significantly reduced on days 4, 7, and 10, with the decrease in full-size-cTnI concentration being more apparent. cTnI/cTnI-C was below 40%. There are three important observations here: The first is that the cTnI-C and full-size-cTnI concentrations were very high at the first diagnosis, indicating that the myocardial injury was serious and acute, and that cTnI was continuously released into the blood; the second is that the full-size-cTnI/cTnI-C ratio was high at the first diagnosis, indicating that cTnI in the blood had not yet been degraded, suggesting that the onset time was short; the third is that the concentrations of cTnI-C and full-size-cTnI were greatly reduced the next day after surgery, but it was still greater than 4 ng/mL. The ratio of full-size-cTnI/cTnI-C was greatly reduced, indicating that the postoperative cTnI in the blood was mainly degraded products, and little newly released cTnI was present in the blood. Thus, the full-size-cTnI/cTnI-C ratio could provide important information for earlier prognoses.

Patient 2, a 79-year-old male, was diagnosed as alcoholic. The cTnI level was slightly higher than normal and no surgical treatment was performed, but the patient was continuously monitored for 3 days. The full-size-cTnI/cTnI-C ratio did not decrease, instead increasing slightly from 74.4 to 82.8%, indicating that the myocardium are continuously damaged by alcoholism, and a small amount of cTnI is continuously released.

Patient 3, a 65-year-old female, was admitted to the hospital due to unconsciousness for 3 h and was diagnosed with acute coronary syndrome and AMI. Although the cTnI concentration was >5 ng/mL, the full-size-cTnI/cTnI-C ratio was below 50%, which indicated that there was less cTnI released into the blood. It was speculated that myocardial damage had been ongoing for some days, and that the patient might be gradually recovering. According to the clinical data, the patent did not undergo surgery during the treatment. Through medical treatment, the condition gradually improved, confirming the speculation.

Patient 4, a 64-year-old male, was diagnosed with AMI. The first recorded cTnI concentration was 6.1 ng/mL. After treatment, the cTnI concentration on the 4th day was 6.4 ng/mL. The prognosis could not be confirmed from the cTnI concentration alone, but the full-size-cTnI/cTnI-C ratio decreased from 67.5% at the first diagnosis and rapidly dropped to 12.5% on the 4th day. The patient was discharged on the 8th day, indicating that combined analysis of the cTnI concentration and full-size-cTnI/cTnI-C ratio allows for earlier prognosis than that afforded by cTnI concentration detection alone.

## Discussion

AMI is an acute ischemic heart disease with a very high mortality rate, and the treatment efficacy and prognosis for AMI are closely related to how soon the treatment begins. Thus, early detection and diagnosis are particularly important. Since cTnI only exists in atrial and ventricular muscles, cTnI cannot enter the blood circulation through the cell membrane when the myocardial cell membrane is intact. When myocardial cells undergo degeneration and necrosis due to ischemia and hypoxia, cTnI can diffuse into intercellular substance through the damaged cell membrane, and then enter blood circulation. Therefore, cTnI is an ideal cardiomyocyte-specific marker that exhibits high specificity and sensitivity to myocardial damage and has high clinical value in the diagnosis, monitoring, treatment efficacy, observation, and prognosis evaluation of AMI, unstable anginapectoris (UAP), perioperative myocardial injury, and other related diseases. Due to the varying duration of chest pain before admission, patient cTnI blood concentrations vary with the duration of the disease. If a dynamic change of cTnI level with time is detected, the specificity of cTnI for the diagnosis of AMI can be improved (10). For patients with acute coronary syndrome (ACS) symptoms, using a >30% relative change in cTnI level combined with the basal concentration or the concentration measured 6 h after hospitalization not only improves specificity, it also effectively assess the risk of cardiac events and death, which clearly has value for the prognosis of patients (11).

At present, the cTnI determination methods used in clinical testing all rely on detecting cTnI fragments protected by cTnC. The fragments are stable and have a long retention time in the blood, which can reflect myocardial damage sensitively (12), but these tests provide no information on the duration of myocardial injury and cannot determine whether it is persistent. Furthermore, patients are reported to present chest pain for some time after admission, which brings greater uncertainty. Since cTnI is released from the myocardium to the blood, it is initially full-length cTnI and is then gradually hydrolyzed. Although full-size-cTnI is easily hydrolyzed, it will be more abundant in the early stages of myocardial injury, so the proportion of full-size-cTnI to total cTnI can be used to indicate the duration since onset. Currently, there are ultra-sensitive methods for detecting cTnI, including CLIA and ELISA (13). However, these methods cannot detect full-size-cTnI and total cTnI simultaneously. In this study, two different tombarthite ions were used to separately label different monoclonal antibodies that recognized the cTnI-stabilized cTnI-C complex and the easily hydrolyzed end region, and thus a method for the simultaneous detection of the cTnI-C complex and full-size-cTnI was established. The fluorescence signal can be amplified by more than one-million times by adding an enhancement solution (14). The sensitivity of this method is very high, reaching 0.02 ng/mL, and the detection wavelengths of different tombarthite ions are different, allowing simultaneous detection of dual indicators cTnI-C-TRFIA and commercial cTnI-CLIA kits were compared, and the correlation coefficient (*R*^2^) of the two methods was found to be 0.9068, i.e., the results were basically the same, indicating that the new method is reliable and that cTnI-C complex concentration can be used to obtain cTnI concentration. Subsequently, using the established full-size-cTnI/cTnI-C-TRFIA, a preliminary retrospective analysis of the serum of several patients was performed as a means to determine full-size-cTnI and cTnI-C and thus calculate the full-size-cTnI/cTnI-C ratio. From the results, the comprehensive analysis of these three indicators was found to provide important information related to the duration of chest pain before admission and the degree of continuous release of cTnI in the myocardium (the acuteness degree of AMI). In addition, it allows earlier prognosis than detecting cTnI alone.

It has been reported that, when studying cTnT, in the early stage of AMI (3–10 h), full-length cTnI-T-C/total cTnI-T-C is around 38.4%. In the late stage (13–30 h) of AMI, this ratio is only 8.9%. Thus, with the onset of AMI, the proportion of full-length cTnI-T-C decreases (8), and our research is also consistent with this conclusion.

In the future, we will use this method for dynamic determination of more samples with the aim of establishing a statistical model based on cTnI-C, full-size-cTnI, and the full-size-cTnI/cTnI-C ratio, which is better for the diagnosis and prognostic evaluation of AMI.

## Data Availability Statement

The raw data supporting the conclusions of this article will be made available by the authors, without undue reservation.

## Ethics Statement

The studies involving human participants were reviewed and approved by The Ethics Committee of the First People's Hospital of Yancheng City. The patients/participants provided their written informed consent to participate in this study. Written informed consent was obtained from the individual(s) for the publication of any potentially identifiable images or data included in this article.

## Author Contributions

BH, JW, HC, PL, and HP contributed to the study concept and design, conducted the literature search, and wrote the manuscript. LZ, PX, and XZ contributed to the data analysis and made the tables and figures. JW, GK, and QW contributed to the collection of patients' samples and medical information. TL, YD, and YW contributed to the acquisition and analysis of data. BH, JW, PL, and HP contributed to the study concept, obtained funding, and critically revised the manuscript. All authors contributed to the article and approved the submitted version.

## Conflict of Interest

The authors declare that the research was conducted in the absence of any commercial or financial relationships that could be construed as a potential conflict of interest.
